# Surface Display of Avian H5 and H9 Hemagglutinin Antigens on Non-Genetically Modified *Lactobacillus* Cells for Bivalent Oral AIV Vaccine Development

**DOI:** 10.3390/microorganisms13071649

**Published:** 2025-07-11

**Authors:** Fuyi Liu, Jingbo Chang, Jingqi Huang, Yuping Liao, Xiaonan Deng, Tingting Guo, Jian Kong, Wentao Kong

**Affiliations:** 1 State Key Laboratory of Microbial Technology, Shandong University, Qingdao 266237, China; 2 School of Life Sciences, Shandong University, Qingdao 266237, China

**Keywords:** *Lactobacillus*, oral vaccine, non-genetically modified organism, surface display

## Abstract

A novel bivalent oral vaccine candidate against H5N1 and H9N2 avian influenza virus (AIV) was developed using *Lactobacillus* surface display technology without genetic modification. The hemagglutinin subunit 1 (HA1) antigens from both subtypes were fused to the surface layer-binding domain of *Lactobacillus crispatus* K313, expressed in *Escherichia coli*, and purified. Wild-type *Lactobacillus johnsonii* H31, isolated from chicken intestine, served as a delivery vehicle by adsorbing and stably displaying the HA1 proteins on its surface. This approach eliminates the need for bacterial engineering while utilizing lactobacilli’s natural capacity to protect surface-displayed antigens, as evidenced by HA1’s protease resistance. Mouse immunization studies demonstrated induction of strong systemic IgG and mucosal IgA responses against both H5N1 and H9N2 HA1. The system offers several advantages, including safety through non-GMO probiotics, potential for multivalent vaccine expansion, and intrinsic antigen protection by lactobacilli. These findings suggest this platform could enable development of cost-effective, multivalent AIV vaccines.

## 1. Introduction

Lactobacilli, as commensal components of the gastrointestinal microbiota, play critical roles in competitive exclusion of pathogens and immunomodulation [1,2,3,4]. Their ability to adhere to intestinal epithelial cells promotes prolonged retention and host-microbe communication, making them attractive candidates for mucosal vaccine delivery. Recent studies have explored *Lactobacillus* strains as live vectors for heterologous antigen presentation [5,6,7,8,9]. Lactobacilli have been demonstrated to enhance mucosal immune responses through enhanced IgA secretion and improved seroconversion, as demonstrated in rotavirus and polio vaccine studies [3]. However, the use of lactobacilli as live vaccine vectors faces challenges in the expression and secretion of certain heterologous proteins, particularly those with structural complexity, which has constrained their wider application in mucosal vaccine development. To overcome these limitations, cell surface display systems using Gram-positive bacterial cell wall anchor domains have been developed [10]. In this approach, target antigens are fused to anchor domains (e.g., N-acetylmuraminidase for peptidoglycan hydrolysis), expressed in *Escherichia coli* (*E. coli*), and subsequently bound to the surface of probiotic lactobacilli. This strategy not only bypasses the need for genetic modification of lactobacilli but also enables the development of multivalent vaccines by co-displaying multiple antigens. Surface (S-) layers are proteinaceous subunits present as the whole cell surface of several species of the genus *Lactobacillus*, as well as in many other bacteria and Archaea. Previous studies have revealed that the C-terminal region of the S-layer protein from *Lactobacillus* is responsible for the cell wall binding [11,12,13,14,15,16], which provides an approach for surface display of heterologous proteins on the cell wall of lactic acid bacteria.

Avian influenza virus (AIV), a significant threat to poultry and public health, is classified into subtypes based on its surface glycoproteins hemagglutinin (HA) and neuraminidase (NA). To date, 18 HA (H1–H18) and 11 NA (N1–N11) subtypes have been identified in avian and mammalian hosts [17,18,19]. HA, synthesized as a precursor (HA0), is cleaved by host proteases into HA1 and HA2 subunits, with HA1 containing the major antigenic epitopes [20,21]. As the primary carrier of receptor-binding domains and antigenic epitopes, the HA1 subunit has become a focal point for vaccine development, diagnostic assays, and antiviral research [22,23,24,25].

In this study, we explored a non-genetically modified, bivalent oral vaccine candidate targeting H5N1 and H9N2 AIV. The HA1 genes of both subtypes were fused to the S-layer binding domain (SBD) of *Lactobacillus crispatus* (*L. crispatus*) K313, expressed in *E. coli*, and purified. The fusion proteins were then anchored to the surface of wild-type *Lactobacillus johnsonii* (*L. johnsonii*) H31—a chicken-derived probiotic strain—eliminating the need for LAB engineering. Mice orally immunized with this formulation produced anti-H5HA1 and anti-H9HA1 serum IgG and mucosal IgA responses, demonstrating the potential of this platform for safe, multivalent AIV vaccine development.

## 2. Materials and Methods

### 2.1. Virus Propagation and Microbial Culture Conditions

The viruses, strains, and plasmids used in this study are listed in Table 1. H5N1 and H9N2 influenza A viruses were propagated in 10-day-old embryonated specific-pathogen-free (SPF) hen’s eggs via allantoic cavity inoculation at 37 °C for 30 h. The virus-containing allantoic fluid was then harvested and stored at −70 °C until further use. For bacterial cultures, *E. coli* strains were grown in LB medium at 37 °C with aeration, supplemented with ampicillin (100 μg/mL) or kanamycin (30 μg/mL) when required. *Lactobacillus* strains were cultured in MRS broth (Oxoid, UK) at 37 °C.

### 2.2. Construction of Expression Plasmids for HA and HA-SBD Fusion Proteins

Viral RNA was extracted from the allantoic fluid using the Axyprep Body Fluid Viral DNA/RNA Miniprep Kit (Axygen Biosciences, Union City, NJ, USA, Cat#AP-MN-BF-VNA-4). First-strand cDNA synthesis was performed by reverse transcription using the universal primer Uni12 (5′-AGCAAAAGCAGG-3′), which is complementary to the conserved 3′ terminus of AIV genomic RNAs [26], with the PrimeScript™ 1st Strand cDNA Synthesis Kit (Takara, Beijing, China, Cat# 6110A). To construct HA1 expression plasmids, the HA1 fragment of the H5N1 subtype was amplified from cDNA using primers H5AF (5′-AAAGTCATATGATTTGCATTGGTTACC-3′; containing an NdeI site) and H5AR (5′-GAGTGGATCCGCCCCCATTGGAGTTTG-3′; containing a BamHI site). The fragment was then cloned into the NdeI/BamHI sites of the pET-22b (+) vector to generate plasmid pET-HA5. Similarly, pET-HA9 was constructed to express the H9N2 HA1 fragment using primers H9AF (5′-GGCCATATGTCAACAAACTCCACAGAAAC-3′; NdeI site) and H9AR (5′-AATGGATCCTTACTTACATTTTGGAATGGC-3′; BamHI site).

The cell wall binding domain (SBD; amino acids 342–501 of SlpB) from *L. crispatus* K313 S-layer protein was cloned into the EcoRI/SalI sites of pET-22b (+), yielding plasmid pET-SBD [14,15]. Fusion expression vectors pET-HA5-SBD and pET-HA9-SBD were constructed by inserting the H5HA1 and H9HA1 fragments, respectively, into the NdeI/BamHI sites of pET-SBD. Notably, neither the HA1 nor SBD genes contained stop codons to enable fusion with the His6-tag encoded by the pET-22b (+) vector.

### 2.3. Expression and Purification of the HA1 and HA1-SBD Proteins in E. coli

For protein expression, plasmids pET-HA5, pET-HA5S, pETHA9, and pET-HA9S were transformed into *E. coli* Origami B (DE3) competent cells. Transformed strains were grown overnight in LB medium at 37 °C, then subcultured (1:100 (*v*/*v*) dilution) into 400 mL of 2 × YT medium and incubated at 37 °C with shaking (200 rpm). When the culture reached an OD_600_ of 0.6, protein expression was induced with 0.1 mM IPTG, followed by incubation at 18 °C for 20 h. The recombinant proteins (H5HA, H5HAS, H9HA, and H9HAS) were purified under native conditions using Ni Sepharose 6 Fast Flow resin (GE Healthcare, Uppsala, Sweden) according to the manufacturer’s protocol. After dialysis against PBS at 4 °C, protein concentrations were determined using the bicinchoninic acid (BCA) assay (Pierce™ BCA Protein Assay Kit, Thermo Fisher Scientific, Rockford, IL, USA). Protein purity and molecular weight were analyzed by SDS-PAGE (8–15% gradient gel) with Coomassie Blue staining.

For Western blot analysis, proteins separated by SDS-PAGE were transferred to PVDF membranes. After blocking with 5% BSA for 1 h at room temperature, membranes were incubated with either mouse anti-HA (H5N1) or anti-HA (H9N2) monoclonal antibodies (eEnzyme, Gaithersburg, MD, USA). Following three washes with TBST (50 mM Tris, 0.1% Tween 20, pH 7.5), membranes were incubated with horseradish peroxidase (HRP)-conjugated goat anti-mouse secondary antibody (Santa Cruz, Dallas, TX, USA) for 1 h. After additional TBST washes, protein bands were visualized using DAB (3,3′-Diaminobenzidine) substrate (10–30 min, room temperature) and rinsed with water to stop the reaction.

### 2.4. Binding of HA-SBD Proteins to Cell Surfaces of Lactobacillus

Both pretreated and untreated cells of *L. crispatus* K313 and *L. johnsonii* H31 were prepared as binding substrates. Stationary-phase bacterial cultures were harvested by centrifugation (5000× *g*, 10 min) and washed once with 0.5 volumes of phosphate-buffered saline (PBS, 137 mM NaCl, 2.7 mM KCl, 10 mM Na_2_HPO_4_, 2 mM KH_2_PO_4_, pH 7.4). For untreated binding substrates, cells were resuspended in PBS to an OD_600_ of 20. For pretreated substrates, cells (1 mL at OD_600_ = 20) were pelleted and incubated with an equal volume of 5% trichloroacetic acid (TCA) at 37 °C for 1 h to modify surface structures [27]. Following treatment, cells were washed twice with PBS before final resuspension in 1 mL PBS.

The binding assays were performed by incubating 20 μL of *Lactobacillus* cells (OD_600_ = 20, approximately 10^9^ cells/mL) with 100 μL of HA or HA-SBD proteins (10 μM, total 1 nmol) for 1 h at room temperature. Following incubation, the cells were pelleted by centrifugation (8000× *g*, 5 min), and the supernatant was carefully collected. The cell pellet was then washed twice with 200 μL of PBS, with each wash supernatant being collected. The combined 500 μL of supernatant (initial and wash fractions) containing unbound protein was quantified using the bicinchoninic acid (BCA) assay (Pierce™ BCA Protein Assay Kit, Thermo Fisher Scientific, Rockford, IL, USA). The amount of bound protein was calculated by subtracting the measured unbound protein from the total input protein (1 nmol).

### 2.5. Immunofluorescence Detection of HA1-SBD on Lactobacillus Cell Surfaces

For immunofluorescence microscopy, *Lactobacillus* cells (10^8^ CFU) pre-incubated with 1 nmol of H5HA1-SBD fusion protein were washed twice with PBS and resuspended in 500 µL of PBS containing 3% BSA (blocking buffer). After blocking at room temperature for 30 min, cells were pelleted by centrifugation (3000× *g*, 5 min) and incubated for 1 h at 37 °C with anti-HA H5N1 mouse monoclonal antibody (1:200 dilution in PBS/3% BSA, eEnzyme, Gaithersburg, MD, USA). Unbound antibodies were removed by three washes with PBS. Cells were then incubated for 1 h at 37 °C with FITC-conjugated goat anti-mouse IgG (1:100 dilution in PBS/3% BSA, Beyotime Biotechnology, Shanghai, China). Following three additional PBS washes, samples were visualized under a Nikon Eclipse 80i fluorescence microscope using FITC filter sets.

### 2.6. Enzymatic Treatment of Surface-Anchored HA1-SBD on Lactobacillus Cells

Surface-bound HA1-SBD on *Lactobacillus* cells (prepared as described above) was subjected to enzymatic digestion with pepsin and pancreatin. For pepsin treatment, cells were incubated in 0.1 M citrate-phosphate buffer (pH 2.8), while pancreatin treatment was performed in PBS (pH 7.4). Both digestions used enzyme concentrations of 0.1% (*w*/*v*) and were carried out at 37 °C for 1 h. Following enzymatic treatment, cells were pelleted by centrifugation (8000× *g*, 5 min) and resuspended in extraction buffer (2% SDS, 1% β-mercaptoethanol) for 10 min at 70 °C to solubilize remaining surface proteins [28]. Parallel control experiments were performed by treating free HA1-SBD protein with the same enzymes under identical conditions. Protein integrity was analyzed by SDS-PAGE followed by Coomassie Blue staining.

### 2.7. Vaccine Preparation and Immunization of Mice

For vaccine preparation, stationary-phase *Lactobacillus* cells (10^9^ CFU) were incubated with 1 mg of HA1-SBD protein at room temperature for 1 h. For bivalent vaccine formulation, equal amounts (0.5 mg each) of H5HA1-SBD and H9HA1-SBD proteins were simultaneously bound to the bacterial cells. After incubation, the protein-bound *Lactobacillus* cells were collected by centrifugation and washed twice with PBS before final resuspension in 500 μL PBS for oral immunization.

All animal procedures were performed in accordance with protocols approved by the Animal Ethics Committee of Shandong University (Protocol No. SYDWLL-2022-002) and complied with the national guidelines for the care and use of laboratory animals. Six-week-old BALB/c mice were divided into five groups with 10 mice per group. Group 1 received *L. johnsonii* H31 bound with H5HA1-SBD, group 2 received *L. johnsonii* H31 with H9HA1-SBD, and group 3 received *L. johnsonii* H31 with both H5HA1-SBD and H9HA1-SBD. Control groups included group 4 (untreated *L. johnsonii* H31) and group 5 (PBS alone). Experimental groups received 200 μL of bacterial suspensions prepared as above (containing ~4 × 10^8^ CFU of *L. johnsonii* H31 bound with SBD-HA1 proteins), while control groups received either 200 μL of unmodified bacteria (~4 × 10^8^ CFU) or PBS alone. Immunizations were administered orally on days 1, 2, 4, 7, 14, and 21.

Two days after the final immunization, mice were sacrificed for sample collection. Blood samples were obtained for serum preparation, while intestinal lavage fluids were collected from ~5 cm of small intestine (10–15 cm distal to the pylorus), which was segmented into 1–2 cm pieces and washed with 1 mL PBS followed by vertexing for 30 s and centrifugation at 8000× *g* for 10 min. All samples were stored at −20 °C until further analysis.

### 2.8. Measurement of Specific Antibodies in Serum and Intestinal Lavage Fluids

HA1-specific serum IgG and intestinal mucosal IgA antibodies were measured by ELISA using purified HA1 antigen as the coating protein. Briefly, 96-well high-binding microtiter plates (Greiner, Frickenhausen, Germany) were coated with 10 µg/mL of either purified H9HA1 or H5HA1 protein in 0.05 M carbonate buffer (pH 9.6) overnight at 4 °C. After washing three times with PBST (PBS containing 0.05% Tween 20), plates were blocked with 5% BSA in PBST at 37 °C for 2 h. Following blocking, plates were incubated with 100 µL of either serum (1:10 dilution in PBST) or intestinal lavage fluid (1:2 dilution in PBST) for 2 h at 37 °C. After thorough washing, 100 µL of horseradish peroxidase (HRP)-conjugated goat anti-mouse IgG (for serum samples) or IgA (for intestinal samples) (Santa Cruz, Dallas, TX, USA; 1:3000 dilution in PBST) was added to each well and incubated for 1 h at 37 °C. After final washes, the reaction was developed by adding 3,3′,5,5′-tetramethylbenzidine (TMB) substrate system for ELISA, and absorbance was measured at 450 nm using a microplate reader.

## 3. Results

### 3.1. Expression and Purification of HA1 and HA1-SBD Proteins in E. coli

**Figure 1:**
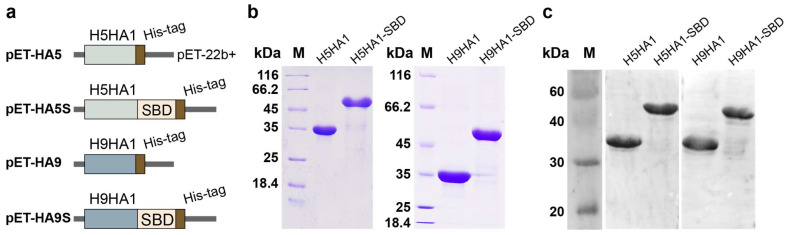
Expression and characterization of HA1 and HA1-SBD recombinant proteins. (**a**) Schematic diagram of HA1 and HA1-SBD fusion protein expression vectors constructed using pET22b+ backbone. (**b**) SDS-PAGE analysis of purified HA1 and HA1-SBD fusion proteins. (**c**) Western blot analysis of whole-cell lysates from *E. coli* expressing HA1 or HA1-SBD fusion proteins, probed with mouse anti-HA monoclonal antibody. The predicted molecular weights for H5HA1, H5HA1-SBD, H9HA1, and H9HA1-SBD are 34.3, 52.79, 34.15, and 52.64 kDa, respectively.

Four recombinant plasmids (pET-HA5, pET-HA9, pET-HA5S, and pET-HA9S) were constructed to express His6-tagged H5N1 HA1, H9N2 HA1, H5N1 HA1-SBD, and H9N2 HA1-SBD proteins, respectively (Figure 1a). Following transformation into *E. coli* Origami B (DE3), the cells were cultured in 2 × YT medium at 37 °C until reaching an OD_600_ of 0.6. Protein expression was then induced with 0.1 mM IPTG at 18 °C for 20 h to maximize soluble protein production. Cells were harvested and lysed by sonication, and the resulting crude extracts were purified using Ni^2+^ affinity chromatography (HisTrap HP, GE Healthcare, Uppsala, Sweden). SDS-PAGE analysis showed that the purified HA1 and HA1-SBD proteins migrated as single bands with apparent molecular masses of approximately 34 kDa and 52 kDa, respectively (Figure 1b).

Western blot analysis of the crude extracts using an HA-specific monoclonal antibody confirmed the immunoreactivity of both HA1 and HA1-SBD proteins (Figure 1c). Distinct bands corresponding to the expected molecular weights were detected, demonstrating that the HA antibody recognized both the native HA1 and the SBD-fused HA1 proteins.

### 3.2. Binding of the Purified Fusion Proteins to the Cell Wall Surface of Lactobacillus

**Figure 2:**
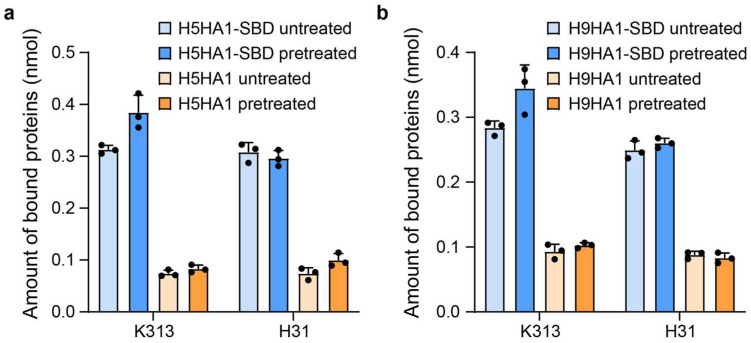
Quantitative analysis of HA1 protein binding to lactobacilli surfaces. (**a**) Binding capacity of *L. crispatus* K313 cells (≈2 × 10^7^ CFU) incubated with 1 nmol H5HA1 or H5HA1-SBD. (**b**) Binding capacity of *L. johnsonii* H31 cells (≈2 × 10^7^ CFU) incubated with 1 nmol H9HA1 or H9HA1-SBD.

The purified fusion proteins (H5HA1, H5HA1-SBD, H9HA1, and H9HA1-SBD) were incubated with *Lactobacillus* cultures following the protocol described in Materials and Methods. Quantitative analysis demonstrated that the HA1-SBD fusion proteins exhibited four-fold greater binding capacity compared to HA1 alone, confirming the SBD domain’s cell wall-binding function (Figure 2). Notably, the binding enhancement showed no significant difference between *L. crispatus* K313 and *L. johnsonii* H31, demonstrating SBD’s cross-strain functionality beyond its original *L. crispatus* source. This broad binding specificity aligns with the known cross-strain functionality of bacterial anchoring domains, as evidenced by reports of AcmA-derived domains from *Lactococcus lactis* binding various lactobacilli and *Bacillus subtilis*, *Lactiplantibacillus plantarum* hydrolase domains functioning across multiple lactobacilli, including *L. johnsonii*, and phage-derived anchors from *Limosilactobacillus fermentum* recognizing diverse lactic acid bacteria [27,29,30]. Further examination of trichloroacetic acid (TCA) pretreatment effects revealed strain-dependent differences: while pretreated *L. crispatus* K313 cells showed higher HA-SBD binding than untreated controls, *L. johnsonii* H31 displayed comparable binding levels regardless of treatment (Figure 2). Under the experimental conditions, binding assays demonstrated that 2 × 10^7^ *Lactobacillus* cells could specifically bind 0.3 nmol of HA1-SBD protein from the initial 1 nmol added, corresponding to approximately 3.6 × 10^4^ HA1-SBD molecules per cell.

**Figure 3:**
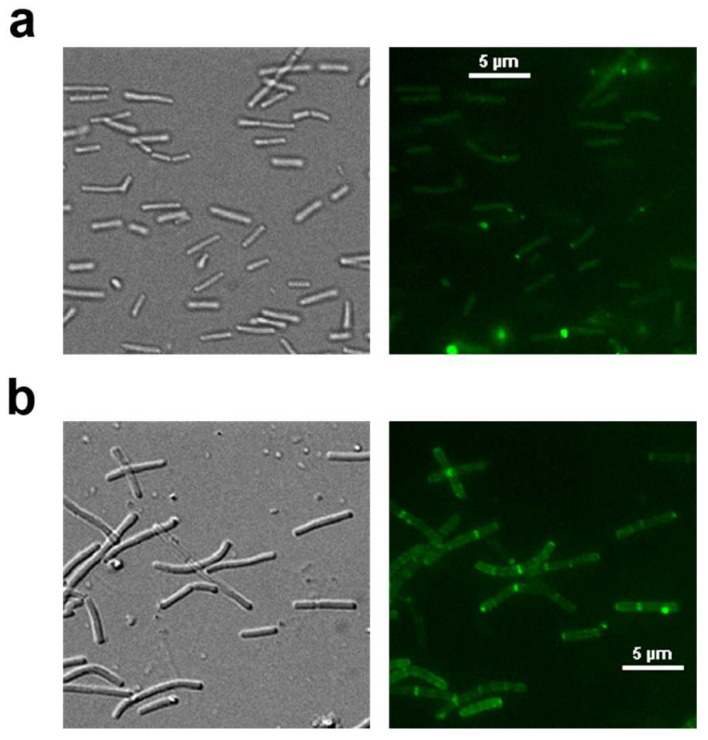
Immunofluorescence analysis of HA1-SBD fusion protein binding to lactobacilli surfaces. (**a**) *L. crispatus* K313 displaying HA1-SBD fusion proteins. Right: FITC fluorescence channel showing antibody-labeled HA1-SBD; Left: Phase-contrast image of the same field. (**b**) *L. johnsonii* H31 displaying HA1-SBD fusion proteins. Right: FITC fluorescence channel; Left: Corresponding phase-contrast image.

Immunofluorescence analysis confirmed both surface localization of HA-SBD fusion proteins and proper exposure of their HA domains (Figure 3). The edge-enhancement pattern, resulting from optical superposition effects at curved cell surfaces, shows stronger peripheral signals (Figure 3b). Additionally, we observed localized fluorescent aggregates that may correspond to active cell wall synthesis sites, where higher concentrations of surface-anchored proteins could accumulate. These features collectively demonstrate effective surface presentation, consistent with established protein display mechanisms in lactic acid bacteria [14,27,29,31,32].

### 3.3. Protease Tolerance of Surface-Displayed Fusion Proteins on Lactobacillus

**Figure 4:**
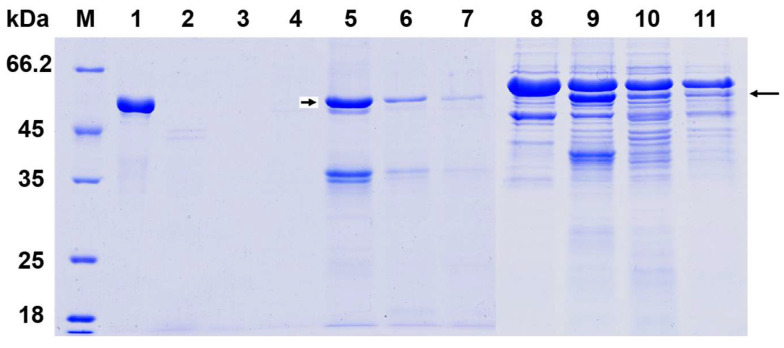
Protease stability analysis of surface-displayed HA1-SBD proteins. The stability of HA1-SBD fusion proteins was evaluated under simulated gastrointestinal conditions. Lane 1 shows untreated H5HA1-SBD control, while lanes 2–3 demonstrate H5HA1-SBD treated with pepsin or pancreatin for 1 h, respectively. Lanes 4–7 display *L. johnsonii* H31 surface proteins: lane 4 shows LiCl-washed control cells, lane 5 after HA1-SBD binding, and lanes 6–7 following subsequent pepsin or pancreatin treatment. Similarly, lanes 8–11 present *L. crispatus* K313 samples under identical experimental conditions, including LiCl-washed control (lane 8), post-binding (lane 9), and after protease treatments (lanes 10–11). Arrows indicate the HA1-SBD bands. All digestions were performed for 1 h at 37 °C.

The protease susceptibility analysis revealed distinct stability patterns between free and cell-bound HA1-SBD proteins. As demonstrated in Figure 4, free HA1-SBD proteins were completely degraded following 1 h treatment with either pepsin (band 2) or pancreatin (band 3), showing no detectable intact protein fragments. In striking contrast, when HA1-SBD was anchored to the cell surface of *L. crispatus* K313 (bands 5–7) and *L. johnsonii* H31 (bands 9–11), the fusion proteins exhibited remarkable protease resistance, with significant portions remaining intact after identical digestive treatments. This protective effect can be attributed to the physical shielding provided by the surface structure of the lactobacilli cell walls, which are likely to limit protease accessibility to the displayed proteins.

The differential stability between free and cell-bound proteins suggests that SBD-mediated surface display not only facilitates protein anchoring but also confers structural protection against gastrointestinal proteases. These findings have important implications for oral vaccine delivery, as the demonstrated resistance to both gastric (pepsin) and intestinal (pancreatin) proteases indicates that SBD-displayed antigens could potentially survive passage through the upper digestive tract and reach the intestinal mucosa intact. The comparable protection observed in both *Lactobacillus* strains further supports the robustness of this display system across different bacterial hosts. This protease-resistant property, combined with the previously demonstrated binding capacity, highlights the potential of this platform for developing effective oral vaccine carriers that can deliver intact antigens to the intestinal mucosa.

### 3.4. HA-Specific IgG and IgA Responses After Oral Administration

**Figure 5:**
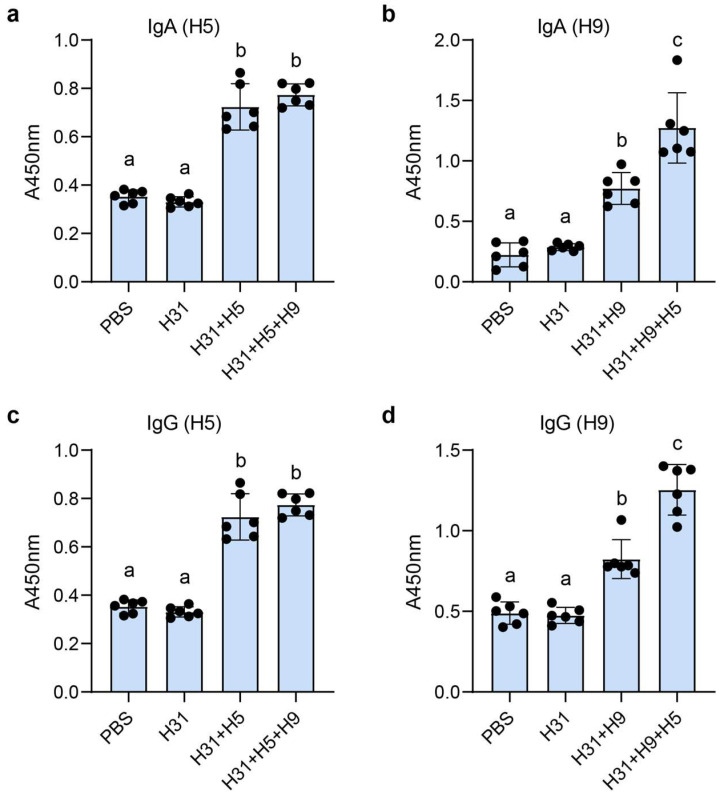
HA1-specific antibody responses in mice following oral immunization. Panels (**a**,**b**) present intestinal IgA levels, while panels (**c**,**d**) show serum IgG levels, as determined by ELISA. For detection, plates were coated with either H5HA1 (**a**,**c**) or H9HA1 (**b**,**d**). Experimental groups included: PBS control, *L. johnsonii* H31 control, monovalent vaccines (H31/H5HA1-SBD or H31/H9HA1-SBD), and bivalent vaccine (H31/H5HA1-SBD + H9HA1-SBD). ELISA results are expressed as relative OD_450_ values. Statistical significance (*p* < 0.02, *t*-test) is indicated by different superscript letters above columns.

Untreated *L. johnsonii* H31 cells were selected for vaccine preparation based on their equivalent HA1-SBD binding capacity compared to TCA-pretreated cells (as shown in Figure 2) and to eliminate potential safety concerns associated with chemical pretreatment methods. Since TCA pretreatment, while enhancing binding in some strains, may pose toxicity risks for in vivo applications, the untreated cells provided an optimal balance of maintained binding efficiency and presumed biosafety. Five experimental groups received either PBS (control), wild-type H31, H5HA1-SBD/H31, H9HA1-SBD/H31, or bivalent H5HA1-SBD + H9HA1-SBD/H31 via oral administration. ELISA analysis using H5HA1- or H9HA1-coated plates demonstrated that sera and intestinal lavage fluids from mice immunized with H5-containing vaccines (H5 and H5 + H9 groups) showed strong reactivity against H5HA1 (Figure 5a,c), while H9 and H5 + H9 groups exhibited significantly higher anti-H9HA1 antibody levels compared to control groups (*p* < 0.02) (Figure 5b,d), confirming successful induction of both systemic IgG and mucosal IgA responses. Interestingly, while anti-H5HA1 antibody levels showed no significant difference between monovalent H5 and bivalent H5 + H9 vaccines, the bivalent formulation elicited notably higher anti-H9HA1 responses than the H9 monovalent vaccine (Figure 5b,d), potentially indicating an adjuvant-like enhancement effect mediated by H5HA1 co-administration. These results collectively demonstrate that SBD-mediated surface display on *L. johnsonii* effectively delivers HA antigens to the gut-associated lymphoid tissue, stimulating robust humoral and mucosal immune responses against both H5 and H9 subtypes, with evidence of potential cross-enhancement in the bivalent formulation. The production of antigen-specific serum IgG and intestinal IgA antibodies confirms the successful mucosal delivery and immunogenicity of this oral vaccine platform.

## 4. Discussion

In this study, we developed an oral vaccine platform by displaying H5N1 and H9N2 HA proteins on *Lactobacillus* cells using the S-layer binding domain of *L. crispatus* K313. Compared to traditional live LAB-based expression systems, this surface display approach offers several advantages, including higher antigen-loading capacity through the use of purified *E. coli*-expressed proteins, avoidance of genetic modification for improved biosafety, and the flexibility to combine multiple antigens for multivalent vaccine design. The choice of cell wall-anchored display is further supported by evidence that surface-exposed antigens tend to be more immunogenic than cytoplasmic or secreted forms [33]. We selected *L. johnsonii* H31, a chicken-derived strain, for oral delivery, as it may function as a potential probiotic and promote immune stimulation [34]. Considering that *L. johnsonii* H31 was originally isolated from chicken intestine and lactobacilli are known to exhibit host-specific colonization [35], this strain appears particularly adapted for poultry vaccine development.

Previous studies indicate that trichloroacetic acid (TCA) pretreatment enhances surface protein binding in some lactic acid bacteria by extracting teichoic acids from the peptidoglycan layer, thereby unmasking hidden binding domains in the cell wall matrix. For instance, TCA treatment significantly improved the display efficiency of α-amylase fused to the P3 cell wall binding domain of *Lactococcus* AcmA peptidoglycan hydrolase [30]. Similarly, enhanced binding of CshA-based fusion proteins was observed in TCA-treated *Limosilactobacillus fermentum* [27]. In our study, while TCA pretreatment moderately increased protein binding in *L. crispatus* K313 (Figure 3), no such enhancement was observed for *L. johnsonii* H31. Considering that PBS washing cannot completely eliminate the potential toxic residues of TCA treatment, we ultimately selected untreated bacterial cells as the safer choice for vaccine delivery applications.

To assess the stability of our vaccine formulation under gastrointestinal conditions, we conducted digestive enzyme tolerance assays, which confirmed that HA1-SBD remained partially bound to lactobacilli after exposure to pepsin and pancreatin. This suggests that the bacterial cell wall provides protective effects, ensuring sufficient antigen delivery to the intestinal mucosa. Following oral immunization in mice, the H5, H9, and bivalent (H5 + H9) vaccine groups all elicited significantly higher levels of anti-HA1 IgG and IgA compared to controls. Notably, the bivalent vaccine induced stronger antibody responses against H9HA than the monovalent version, suggesting potential advantages of multivalent vaccine formulations.

Compared to conventional inactivated whole-virus vaccines widely used in poultry [36], our *Lactobacillus*-based platform demonstrates several key advantages, including enhanced safety through complete avoidance of viral components, stress-free oral delivery, and modular design allowing simultaneous presentation of H5/H9 antigens with potential for further expansion. Additionally, the live probiotic vector may provide ancillary gut health benefits. Unlike recombinant *Lactobacillus* strains secreting antigens [37], our surface-display system not only circumvents the technical challenges of difficult-to-secrete antigens but also achieves higher and more consistent antigen loading without genetic modification requirements. However, the current surface-display system relies on exogenously anchored rather than bacterially secreted antigens, resulting in shorter intestinal retention compared to secretion-based systems and likely necessitating multiple immunizations for optimal efficacy. Additionally, the dependence on purified *E. coli*-expressed antigens contributes to higher production costs. Future work will explore cost-reduction strategies, with direct use of *E. coli* lysates for antigen coupling as a potential solution.

In conclusion, our study establishes an effective oral vaccine platform using non-GMO lactobacilli with SBD-mediated surface display technology. This system successfully induced specific antibody responses against avian influenza HA proteins in mice, demonstrating both strong immunogenicity and gastrointestinal stability. The findings provide a safe, cost-effective mucosal vaccine strategy with significant potential for poultry vaccination against avian influenza and other mucosal pathogens.

## Figures and Tables

**Table 1:** Virus, strains, plasmids used in this study.

Material	Relevant Features	Source or Reference
**Virus**		
Influenza A virus (A/chicken/Shandong/ K01/2004(H5N1))	Used for cloning hemagglutinin gene	This study
Influenza A virus (A/chicken/Shandong/ K02/2004(H9N2))	Used for cloning hemagglutinin gene	This study
**Strains**		
*E. coli* DH5α	Cloning host	TaKaRa
Origami B (DE3)	Expression host	Novagen
*Lactobacillus crispatus* K313	Used for cloning the S-layer protein and binding the antigen	[15]
*Lactobacillus johnsonii* H31	Used for binding the antigen	[15]
**Plasmids**		
pET-22b (+)	*E. coli* expression vector, T7 promoter, Amp^R^	Novagen
pET-SBD	pET2209; pET-22b (+) carrying S-layer protein cell wall binding domain (from 342 aa to 501 aa) of *Lactobacillus crispatus* K313; expressing His6-tagged SBD	[15]
pET-HA5	pET-22b (+) vector containing H5HA1 gene inserted between NdeI and BamHI restriction sites for expression of His6-tagged H5HA1 protein	This study
pET-HA5S	H5HA1 was inserted into NdeI/BamHI sites of pET-SBD to express His6-tagged H5HA1-SBD fusion protein	This study
pET-HA9	pET-22b (+) vector containing H9HA1 gene inserted between NdeI and BamHI restriction sites for expression of His6-tagged H9HA1 protein	This study
pET-HA9S	H9HA1 was inserted into NdeI/BamHI sites of pET-SBD to express His6-tagged H5HA1-SBD fusion protein	This study

## Data Availability

The original contributions presented in this study are included in the article. Further inquiries can be directed to the corresponding authors.
